# Protective effect of DLX6-AS1 silencing against cerebral ischemia/reperfusion induced impairments

**DOI:** 10.18632/aging.104070

**Published:** 2020-11-18

**Authors:** Xiamin Hu, Zifei Xiang, Wei Zhang, Zhijun Yu, Xiaoming Xin, Rong Zhang, Youping Deng, Qiong Yuan

**Affiliations:** 1 College of Pharmacy, Shanghai University of Medicine and Health Sciences, Shanghai, China; 2 Institute of Pharmaceutical Innovation, Hubei Province Key Laboratory of Occupational Hazard Identification and Control, College of Medicine, Wuhan University of Science and Technology, Wuhan, Hubei Province, China; 3 China Resources and WISCO General Hospital, Wuhan, Hubei Province, China; 4 Bioinformatics Core, Department of Complementary and Integrative Medicine, University of Hawaii John A. Burns School of Medicine, Honolulu, HI 96813, USA

**Keywords:** DLX6-AS1, miR-149-3p, Bcl-2-related ovarian killer, BOK, stroke

## Abstract

In the present study, we investigated the role of lncRNA mus *distal-less homeobox 6 antisense 1 (DLX6-AS1)* during cerebral impairment induced by stroke. DLX6-AS1 levels were upregulated during ischemia/reperfusion (I/R) and downregulation of DLX6-AS1 reduced acute injury and ameliorated long-term neurological impairments induced by cerebral I/R in mice. Additionally, silencing of DLX6-AS1 significantly decreased the neuronal apoptosis in vivo and in vitro. Furthermore, inhibition of miRNA-149-3p led to enhance the apoptosis, which confirmed that DLX6-AS1 could sponge miR-149-3p. Finally, BOK was predicted to be the target of miR-149-3p using TargetScanVert software. And the silencing of DLX6-AS1 inhibited BOK expression both *in vivo* and *in vitro*, which was reversed by a miR-149-3p inhibitor. At meantime, BOK promoted OGD/R induced apoptosis in N2a cells. Therefore, this suggests that miR-149-3p sponging by DLX6-AS1 may lead to cerebral neuron I/R-induced impairments through upregulation of apoptotic BOK activity, which offers a new approach to the treatment of stroke impairment.

## INTRODUCTION

Stroke is one of the foremost causes of disability and is responsible for many premature deaths worldwide [1]. The development of successful treatments for stroke has been restricted for a number of reasons because first, brain injury produced by ischemia develops rapidly, second, the complex interplay between the signaling pathways involved and third, the brief treatment window aimed at particular targets [1]. Therefore, identification of an effective treatment target would be beneficial for the treatment of stroke.

At least 90% of the human genome is transcribed into RNA but < 2% has been shown to encode proteins [2]. Long non-coding RNAs (lncRNAs) represent one type of ncRNAs, while transfer RNAs (tRNAs), small nucleolar RNAs (snoRNAs) and small-interfering RNA (siRNAs) are the other main types of RNAs that are non-coding. LncRNAs are believed to play vital roles during cellular differentiation and development, as well as in many mechanisms including modulation of proliferation and invasiveness of tumors and the reprogramming of pluripotent stem cells that have been previously induced [3, 4]. *Distal-less homeobox 6 antisense 1 (DLX6-AS1)*, with a length of 1,990 bps, represents a developmentally regulated lncRNA [5]. DLX6-AS1 has been shown to have a pivotal role in pre-eclampsia by modulating trophoblast migration and invasion via the miR-376c/growth arrest and DNA damage inducible alpha (GADD45A) pathway [6]. According to genotype-tissue expression (GTEx) analysis, DLX6-AS1 is transcribed in high levels in the normal brain, but up to now no evidence has been presented of a role for DLX6-AS1 in stroke.

MicroRNAs (miRNAs), another type of ncRNAs, are short small non-coding RNA molecules with a length of 20–24 bps and act to inhibit target mRNA translations [7]. Several research groups have reported that several miRNAs are involved in ischemia-induced progressive brain impairment induced by a variety of target genes [8, 9]. Recent research has confirmed that *miR-1247-3p* regulates apoptosis of neurons elicited by stroke [10]. One of the primary mechanisms of lncRNAs is that of a competing endogenous RNA (ceRNA) in order to regulate mRNA transcription via competing for shared microRNAs (miRNAs).

Through bioinformatic analysis, DLX6-AS1 has been shown to bind to miR-149-3p. It has been reported that *miR-149-3p* is involved in inhibiting adipogenic differentiation of bone marrow-derived mesenchymal stem cells [11]. Additionally, reduced miR-149-3p expression has been proven to significantly improve whole-body insulin sensitivity and decrease subcutaneous adipose tissue inflammation and liver steatosis in high-fat fed mice. However, the potential effect of miR-149-3p on cerebral impairment remains to be established.

*BOK* governs the intrinsic apoptotic pathways [12] and is known to possess 3 BCL-2 homology domains. It has been proposed to act on the same pro-apoptotic pathway as a number of pro-apoptotic proteins (e.g. BAK, BAX) [13]. BOK can serve as a prognostic marker for colorectal cancer, and it has been suggested that different levels of BOK activity drives the progression of cancer. TargetScanVert software analysis has predicted that BOK may be a gene targeted by miR-149-3p. Therefore we hypothesized that BOK might play a role in stroke-induced cerebral neuronal apoptosis and that miR-149-3p regulates BOK expression.

Thus, our aims were to investigate the potential actions of DLX6-AS1 in cerebral I/R-induced neuron injury and to identify whether the miR-149-3p/BOK signaling pathway regulates the function of DLX6-AS.

## RESULTS

### Silencing of DLX6-AS1 reduced acute injury of cerebral neurons

Mice were treated with cerebral ischemia (IS) / reperfusion (R) on day 7 after being injected icv with lentivirus DLX6-AS1-shRNA1, and then DLX6-AS1 expression was first measured in the ischemic cerebral hemisphere. As shown in Supplementary Figure 1A, DLX6-AS1 was upregulated after ischemia for 1 h (IS 1 h) and following reperfusion for 24 h (R 24 h) (1.72 ± 0.19 arbitrary units (a.u.)) compared with that of the sham group (1.04 ± 0.04 a.u., *P* < 0.05). To establish the actions of DLX6-AS1 after cerebral I/R, the expression of DLX6-AS1 of the mouse brain should been interrupted. There were three interrupted sequences were designed and transfected into N2a cells. The results showed that DLX6-AS1 shRNA1 played the best interrupted effect on DLX6-AS1 expression (Supplementary Figure 1B, the results of other two shRNA were not shown here). Therefore, DLX6-AS1 shRNA sequence was chose for the further experiment. The mouse was injected icv with LV-DLX6-AS1-shRNA or LV-NEG on day 7 before cerebral I/R. and then neurological functions were evaluated in Morris water maze and pole tests from day 22 through day 28 after cerebral I/R. In comparison to the LV-NEG group, DLX6-AS1 expression was successfully downregulated (0.29 ± 0.06 a.u., Supplementary Figure 1A) after icv injection with LV-DLX6-AS1-shRNA. Behavioral assessments were used to assess neurological functions and TTC staining to determine the volume of each lesion.

**Figure 1:**
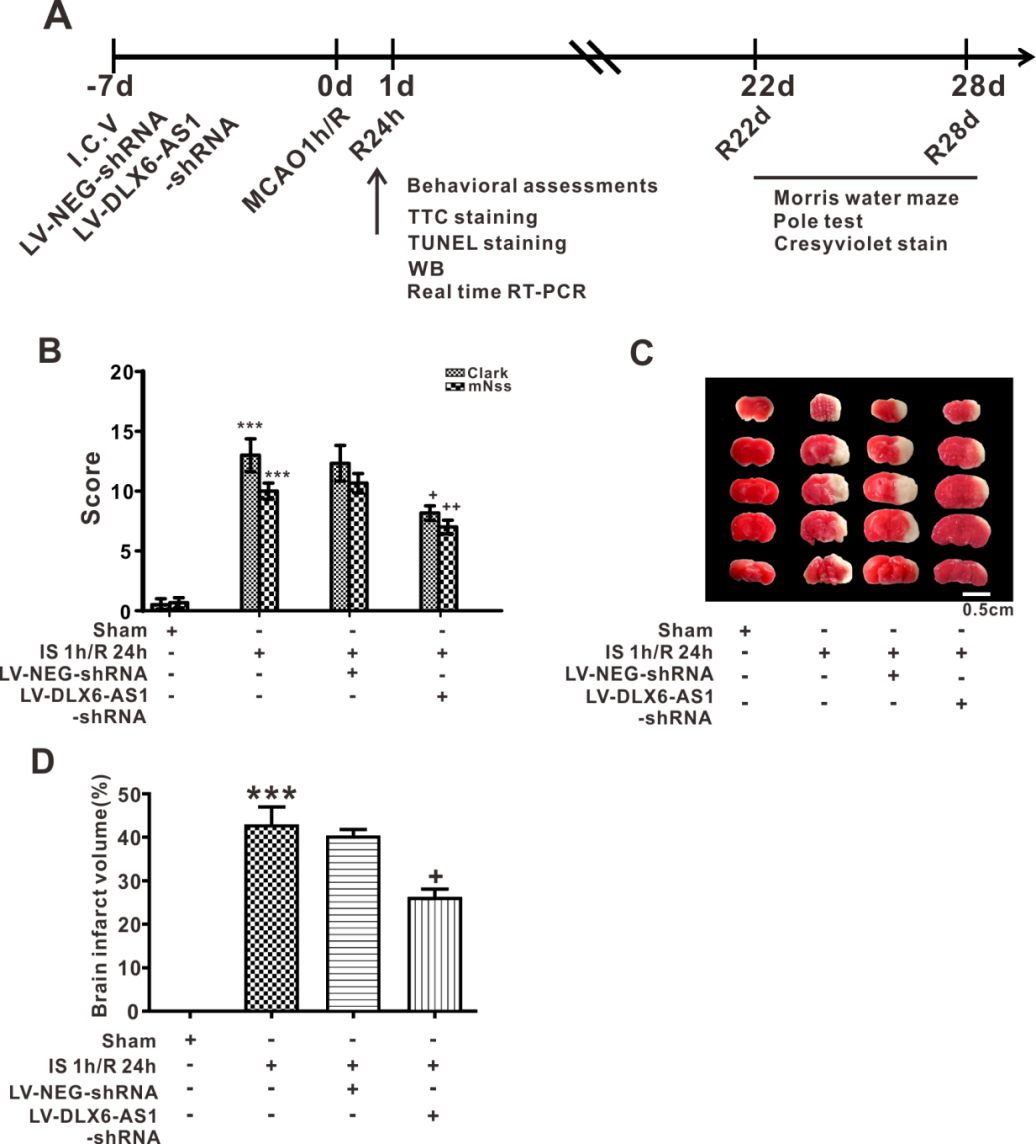
**DLX6-AS1 silencing inhibited the acute injury of cerebral neurons induced by brain ischemia/reperfusion.** (**A**) Flow chart of the study investigating DLX6-AS1 function. (**B**) Average neurological scores of the Clark and mNSS tests. (**C**) Representative images and (**D**) Statistical chart for TTC staining. Values represent the mean ± SEM (n = 6 for each group). ***P < 0.001 vs sham; +P < 0.05, ++P < 0.01 vs IS 1 h/R 24 h + LV-NEG.

According to the results of mNSS, the scores of ischemia in the IS 1 h/R 24 h group (10.00 ± 0.68) were greater than in the sham animal group (0.67 ± 0.42, *P* < 0.001, Figure 1B). However, when the DLX6-AS1 level was reduced, the score of the LV-DLX6-AS1-shRNA+IS 1 h / R 24 h group was lower than the LV-NEG+IS 1 h / R 24 h group. Similar results were found using Clark scoring (Figure 1B). Following TTC staining, we found that there was a more significant brain infarct in the IS 1 h / R 24 h group in comparison to the sham group and that silencing of DLX6-AS1 expression reduced the infarct volume in the IS 1 h / R 24 h group (Figure 1C).

### Silencing of DLX6-AS1 ameliorated long-term neurological impairments induced by cerebral ischemia/reperfusion in C57/BL6 mice

**Figure 2:**
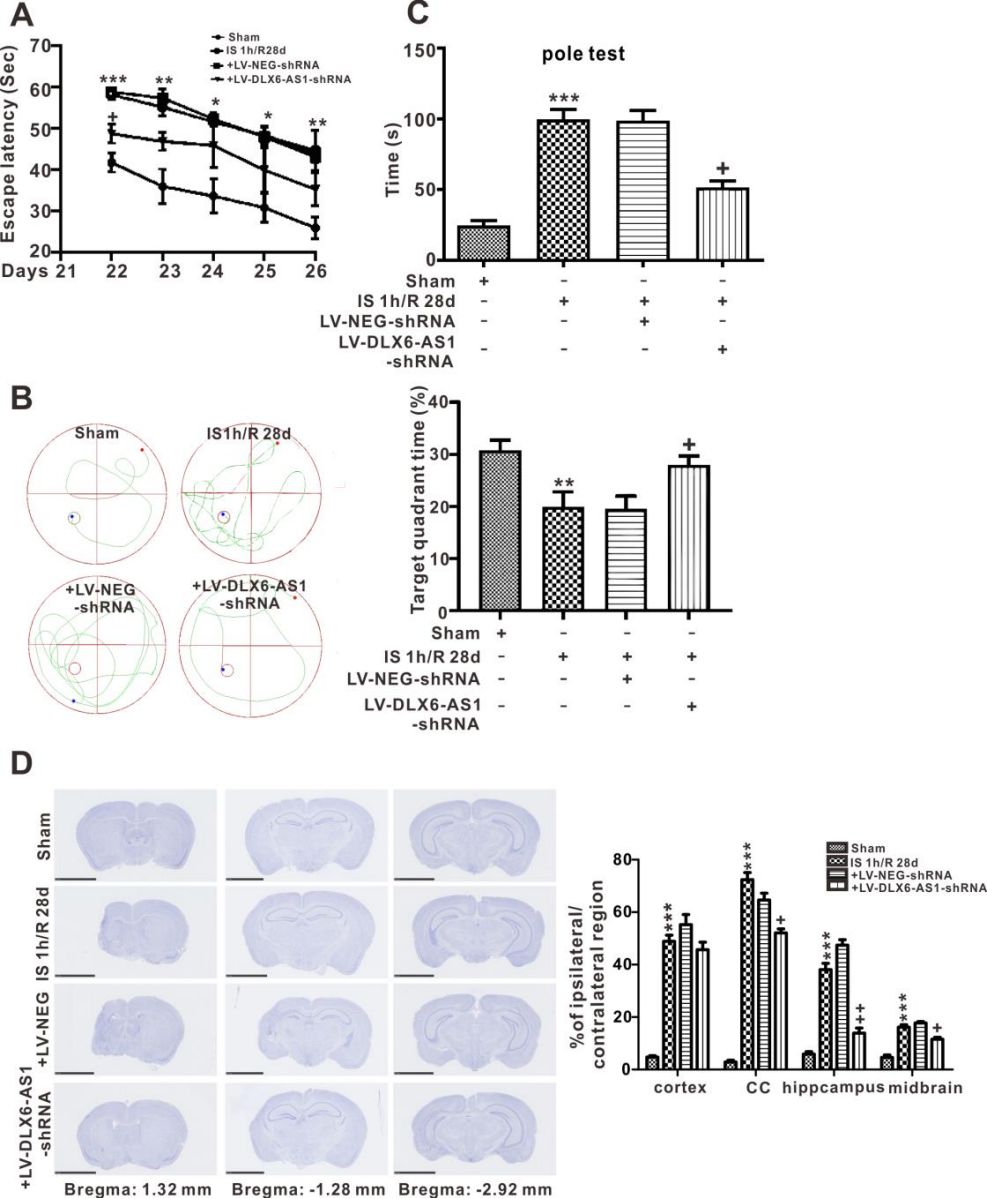
**DLX6-AS1 silencing ameliorated long-term neurological dysfunction after ischemia.** (**A**) the escape latency, (**B**) representative image and statistical evaluation of the Morris water maze and the target quadrant time tests (**C**) the pole test, (**D**) photomicrograph (cresyl violet stain) and statistical chart showing the regions of the cortex, corpus callosum, hippocampus, and midbrain of the brain. Values represent mean ± SEM (n = 6 in each group). *P < 0.05, **P < 0.01, ***P < 0.001 vs sham; +P < 0.05, ++P < 0.01 vs + LV-NEG-shRNA.

To investigate further the long-term effects of DLX6-AS1 in cerebral I/R, the Morris water maze test was utilized to assess impairments in memory function and pole tests were employed to evaluate motor functions, while cresyl violet staining was used to investigate brain atrophy. The latencies of escape of all mice groups exhibited downward trends with experience. In addition, there were significant changes in escape latencies between the DLX6-AS1-down+IS 1 h/R 24 h and the IS 1 h/R 24 h groups during reperfusion from day 22 through day 26. However, the IS 1 h/R 24 h group at day 28 showed a marked retardation in their escape latencies compared to the sham group (Figure 2A). The movement trajectories of the mice in each group were also measured. The time spent in the second quadrant (Figure 2B) was shorter in the IS 1 h/R 24 h group at day 26 than in the other 3 groups (*P* < 0.05, Figure 2B), which perhaps indicated that cerebral I/R induced memory deficits had occurred.

The motor functions of the experimental mice were assessed using the pole test. The total time a mouse spent turning and moving to the bottom of the rod was greater in the IS/R group (Figure 2C, *P* < 0.001). In comparison solely with the IS/R group, silencing of DLX6-AS1 significantly reduced the total time in the IS/R plus LV-DLX6-AS1-shRNA group. To determine whether silencing of DLX6-AS1 could suppress regional brain atrophy, the volumes of atrophic regions were measured (percentage of contralateral hemisphere). Extreme alterations in the cerebral IS/R were readily found in the hippocampus and striatum compared to with the sham-operated group, and these changes were alleviated by treatment with LV-DLX6-AS1- shRNA (cc: 53.2% ± 2.1%, *P* < 0.05; hippocampus: 15.9% ± 2.4%, *P* < 0.01; midbrain: 12.6% ± 1.1%; Figure 2D).

### Silencing of DLX6-AS1 inhibited neuronal apoptosis

**Figure 3:**
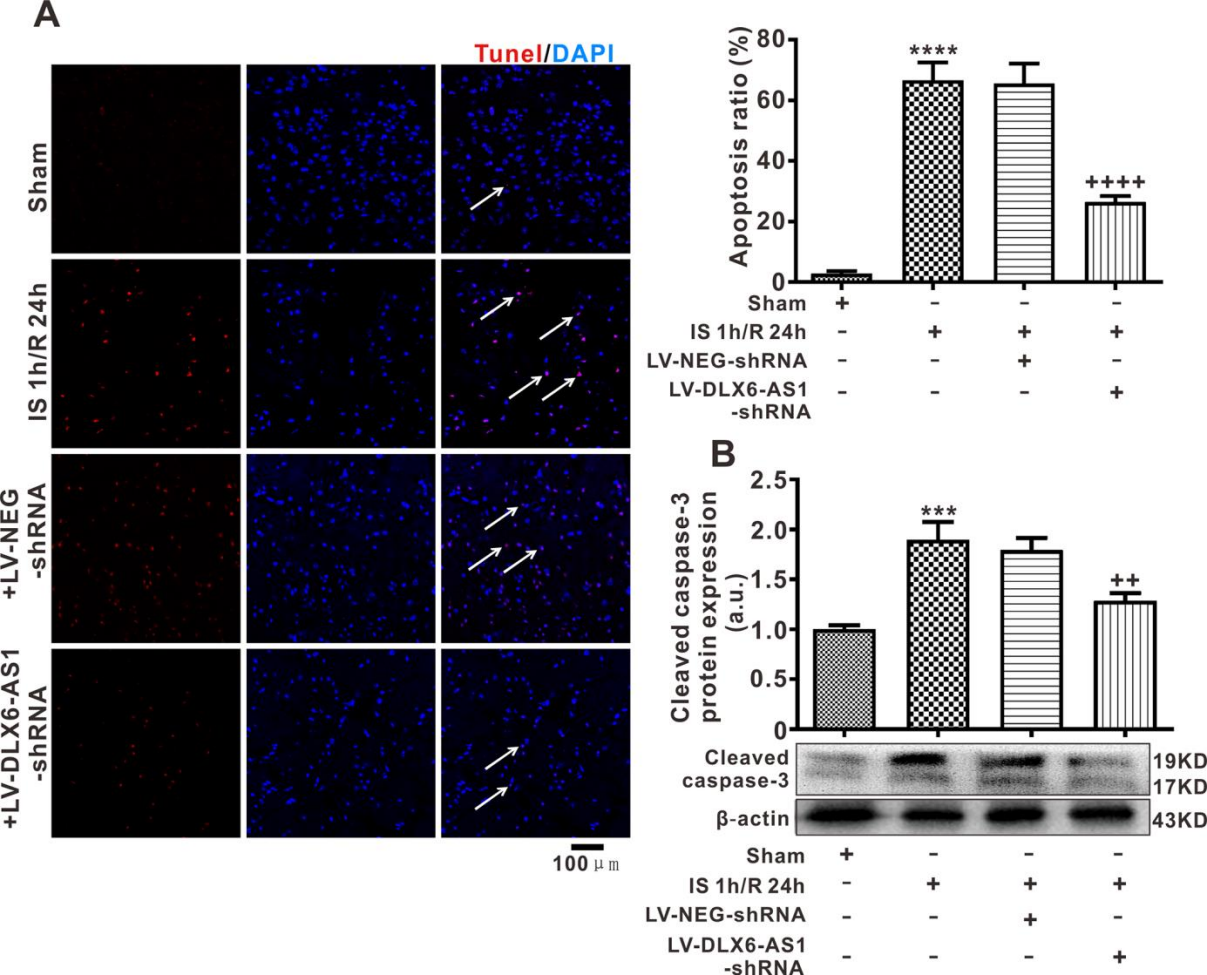
**DLX6-AS1 silencing inhibited neuronal apoptosis induced by brain ischemia/reperfusion.** (**A**) Representative images and statistics of TUNEL staining of brain sections for assaying neuronal apoptosis. (**B**) Cleaved caspase-3 protein expression, measured by western blotting. Data are given as the mean ± SEM (n = 6 in each group). ***P < 0.001 vs sham; ^++^P < 0.01, ^+++^P <0.001 vs IS 1 h/R 24 h + LV-NEG-shRNA.

To detect the actions of DLX6-AS1 on neuronal apoptosis triggered by cerebral I/R, TUNEL staining and the cleaved expression of caspase-3 protein in ischemic cerebral hemispheres were determined *in vivo*. In comparison with the sham-operated group of mice, the ratios of neuronal TUNEL-positive cells (44.7 ± 4.5%, *P* < 0.001) and cleaved caspase-3 (1.87 ± 0.18, *P* < 0.01) were significantly higher in the IS 1 h/R 24 h group (Figure 3A, 3B). However, the ratio of TUNEL-positive cells and cleaved caspase-3 protein in the LV-DLX6-AS1-shRNA+IS 1 h/R 24 h group was significantly lower in comparison with solely the IS 1 h/R 24 h group (Figure 3A, 3B). These data revealed that silencing of DLX6-AS1 inhibited neuronal apoptosis induced by brain ischemia/reperfusion.

### Silencing of DLX6-AS1 reduced apoptosis of N2a and SH-SY5Y cell lines induced by OGD/R

In order to establish further the effects of DLX6-AS1 *in vitro*, N2a and SH-SY5Y cells were treated with OGD/R. According to the RT-PCR results, DLX6-AS1 was upregulated time-dependently in both cell lines (Supplementary Figure 2A). Additionally, DLX6-AS1 was at its highest level (3.00 ± 0.36) after OGD 3 h and following reoxygenation at 24 h compared with the control group (untreated cells) (1.15 ± 0.24, *P <* 0.01, Supplementary Figure 2A). Additionally, upregulation of DLX6-AS1 expression occurred in a time-dependent fashion in SH-SY5Y cells (Supplementary Figure 2B). DLX6-AS1 expression was increased compared to the controls (1.08 ± 0.13, Supplementary Figure 2B) after OGD 3 h and following reoxygenation for 12 h (2.40 ± 0.04, *P <* 0.01) or 24 h (1.85 + 0.16, *P <* 0.05), in comparison to controls (1.08 ± 0.13, Supplementary Figure 2B).

**Figure 4:**
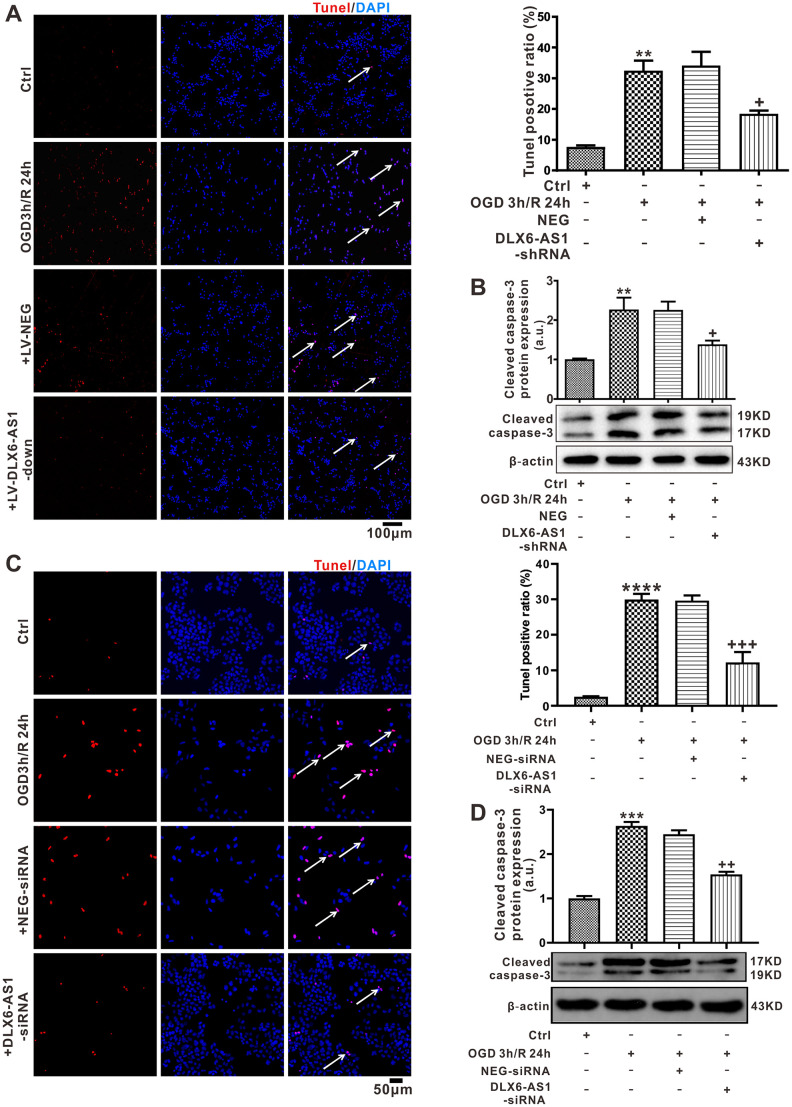
**DLX6-AS1 was upregulated in an OGD/R model and DLX6-AS1 silencing reduced apoptosis of N2a and SH-SY5Y cells induced by OGD/R.** (**A**) Representative images and statistics of TUNEL staining of N2a cells used to confirm apoptotic changes (100X). (**B**) Cleaved caspase-3 protein levels measured by western blotting. (**C**) Representative images and statistics of TUNEL staining of SH-SY5Y cells used to confirm apoptotic changes (100X). (**D**) Cleaved caspase-3 protein levels of SH-SY5Y measured by western blotting. Values represent mean ± SEM (n = 3 in each group). **P < 0.01, ***P < 0.001 vs Ctrl; +P < 0.05, ^++^P < 0.01, ^+++^P < 0.001 vs OGD 3 h/R 24 h + NEG.

To confirm the actions of DLX6-AS1 on neuronal apoptosis, N2a cells were transfected with DLX6-AS1-shRNA for 48 h and then exposed to OGD for 3 h; followed by 24 h re-oxygenation. Apoptotic cell numbers (32.48% ± 3.26%) and expression levels of cleaved caspase-3 (2.27 ± 0 .30) elicited by OGD/R were greater than for untreated control cells (7.64% ± 0.56%, 1.01 ± 0.02, Figure 4A, 4B). Downregulation of DLX6-AS1 levels with DLX6-AS1-shRNA significantly decreased cell apoptosis (18.48 ± 1.03) and cleaved caspase-3 expression (1.38 ± 0.10) induced by OGD/R (Figure 4A, 4B). Consistent with the N2a cells finding, transduction of SH-SY5Y cells with DLX6-AS1 siRNA (Supplementary Figure 2B) significantly reduced apoptotic cell numbers and decreased the expression of apoptotic proteins cleaved caspase-3 (Figure 4C, 4D) to inhibited OGD-R-induced apoptosis. The data indicated that silencing of DLX6-AS1 also inhibited neuronal apoptosis induced by OGD/R *in vitro*.

### miR-149-3p as a potential target for DLX6-AS1

**Figure 5:**
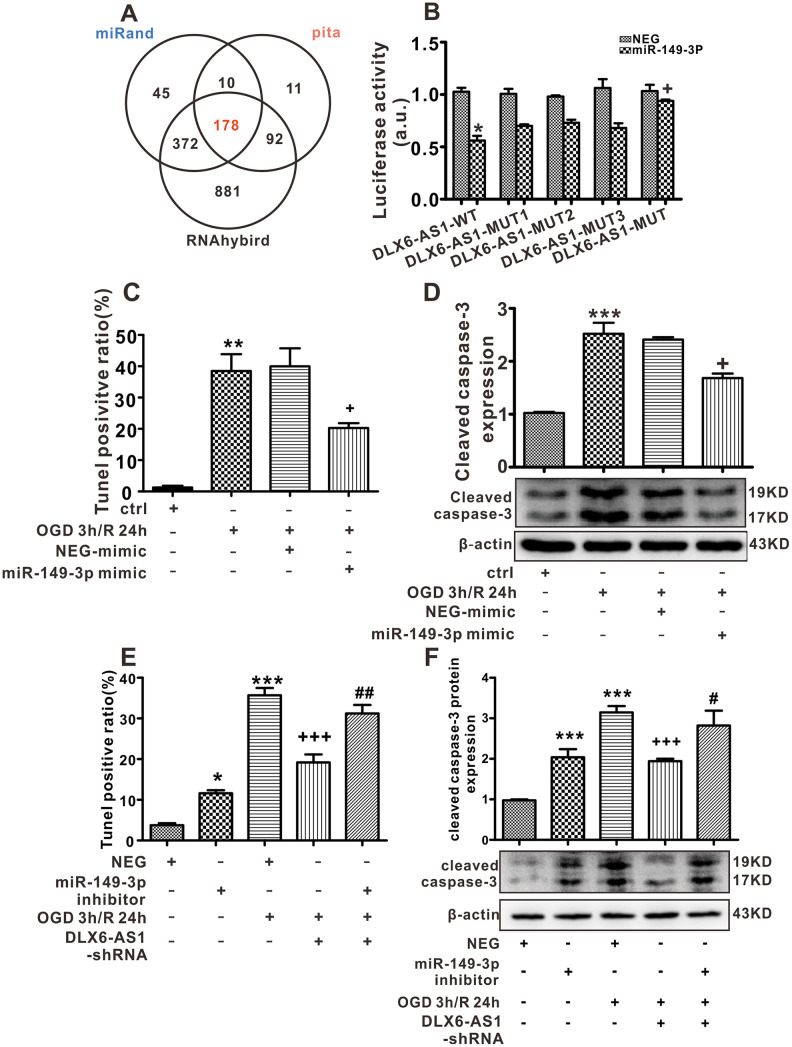
**miR-149-3p may be a target of DLX6-AS1.** (**A**) Predicted binding miRNAs of DLX6-AS1 derived from 3 databases. (**B**) Luciferase assay results of DLX-AS1 wild type (WT) and mutant (MUT) construct binding with mmu-miR-149-3p. (**C**) Effects of a miR-149-3p mimic on apoptosis induced by OGD/R, as detected by TUNEL staining. (**D**) Effects of a miR-149-3p mimic on caspase-3 expression induced by OGD/R in N2a cells detected by western blotting. (**E**) Effects of a miR-149-3p inhibitor on apoptosis induced by OGD/R, as detected by TUNEL staining. (**F**) Effects of a miR-149-3p inhibitor on caspase-3 expression induced by OGD/R in N2a cells detected by western blotting. Values represent the mean ± SEM (n = 3 in each group). *P < 0.05, ***P < 0.001 vs NEG, **P < 0.01 vs Ctrl; ^+^P < 0.05 vs OGD 3 h/R 24h + NEG-mimic, ^+++^P < 0.001 vs OGD 3 h/R 24 h + NEG; ^#^P < 0.05, ^##^P < 0.01 vs OGD 3 h/R 24 h + DLX6-AS1-shRNA.

In order to investigate the mechanism of DLX6-AS1 effects, the databases of pita, miRanda and RNAhybrid were interrogated to look for a predicted possible target miRNA that DLX6-AS1 might act on as a ceRNA. As shown in Figure 5A, 180 miRNAs in total were predicted in the 3 databases examined. Interestingly, miR-149-3p exhibited high scores (Table 1), and the scores of mmu-miR-149-3p and hsa-miR-149-3p were surprisingly similar (Table 1, Supplementary Figure 3A). Hence, we speculate that DLX6-AS1 acts as a ceRNA and competes with miR-149-3p. Therefore, we set out to detect the DLX6-AS1 sponging ability of miR-149-3p. By analyzing bioinformatics data, it was established that DLX6-AS1 has 3 sites that can bind to miR-149-3p. Therefore, wild-type and mutant forms of DLX6-AS1 were constructed. The miR-149-3p bound to all 3 sites of DLX6-AS1. Mutants of DLX6-AS1 separately abolished the binding effects of DLX6-AS1 on miR-149-3p, activity detected by luciferase assays (Figure 5B).

**Table 1:** The binding miRNA of DLX6-AS1.

**miRNA**	**miRanda score**	**Pita ddG**	**RNAhybrid MFE**
mmu-miR-130a-3p	141	-10.79	-21
mmu-miR-9-5p	152	-10.96	-22
mmu-miR-135a-1-3p	298	-14.45	-27.7
mmu-miR-149-3p	145	-18.34	-34.7
mmu-miR-150-5p	156	-12.67	-27.2
mmu-miR-155-3p	158	-11.53	-26
mmu-miR-181a-5p	309	-12.04	-24.1
mmu-miR-188-5p	145	-15.02	-28.2
mmu-miR-195a-3p	307	-14.78	-28
mmu-miR-202-3p	292	-11.44	-25.8

Furthermore, miR-149-3p expression levels were determined both *in vivo* and *in vitro* and it was demonstrated that miR-149-3p levels were abridged in mice brains treated with IS 1 h/R 24 h (Supplementary Figure 3B). In addition, the smallest concentrations of miR-149-3p were revealed 12 h after reperfusion of the *in vitro* N2a cells OGD/R test (0.51 ± 0.039, *P <* 0.01) compared to control mice (Supplementary Figure 3C), which indicated an opposite pattern to that of DLX6-AS1 expression. To investigate further the relationship between miR-149-3p and DLX6-AS1, we constructed miR-149-3p mimic and inhibitor sequences. After transfection with an miR-149-3p mimic for 48 h, miR-149-3p was successfully overexpressed (31.82 ± 4.24, *P <* 0.001, Supplementary Figure 3D).

The data from TUNEL staining and western blotting revealed that when miR-149-3p was overexpressed there was a significant reduction of apoptosis in OGD/R N2a cells (ratio of TUNEL-positive cells: 20.25% ± 1.57%, cleaved caspase-3: 1.68 ± 0.09) compared to NEG+OGD 3 h/R 24 h cells (ratio of TUNEL-positive cells: 39.99% ± 5.76%, cleaved caspase-3: 2.41 ± 0.04; Figure 5C, 5D). However, after transfection with an miR-149-3p inhibitor, the degree of cellular apoptosis (the ratio of TUNEL-positive cells: 11.63% ± 0.73%, cleaved caspase-3: 2.04 ± 0.20) was increased in comparison to the NEG group (i.e., the ratio of TUNEL-positive cells: 3.75% ± 0.513%, cleaved caspase-3: 0.97 ± 0.02, Figures 5C, 5D). As previously demonstrated, silencing of DLX6-AS1 reduced the degree of apoptosis of neurons elicited by OGD/R. It is noteworthy that in cells transfected with DLX6-AS1-shRNA and miR-149-3p inhibitors exposed to OGD/R (the ratio of TUNEL-positive cells: 31.21% ± 2.11%, cleaved caspase-3: 2.82 ± 0.37), apoptosis increased more than in the DLX6-AS1-shRNA+OGD 3-h/R 24-h group (ratio of TUNEL-positive cells: 19.17% ± 1.98%, *P* < 0.01; cleaved caspase-3: 1.94 ± 0.06, *P <* 0.05, Figure 5E, 5F). The data showed that a miR-149-3p inhibitor reversed the anti-apoptotic effect of DLX6-AS1-shRNA in cells treated with OGD/R, indicating that miR-149-3p may well be a target of DLX6-AS1.

### The target of BOK

**Figure 6:**
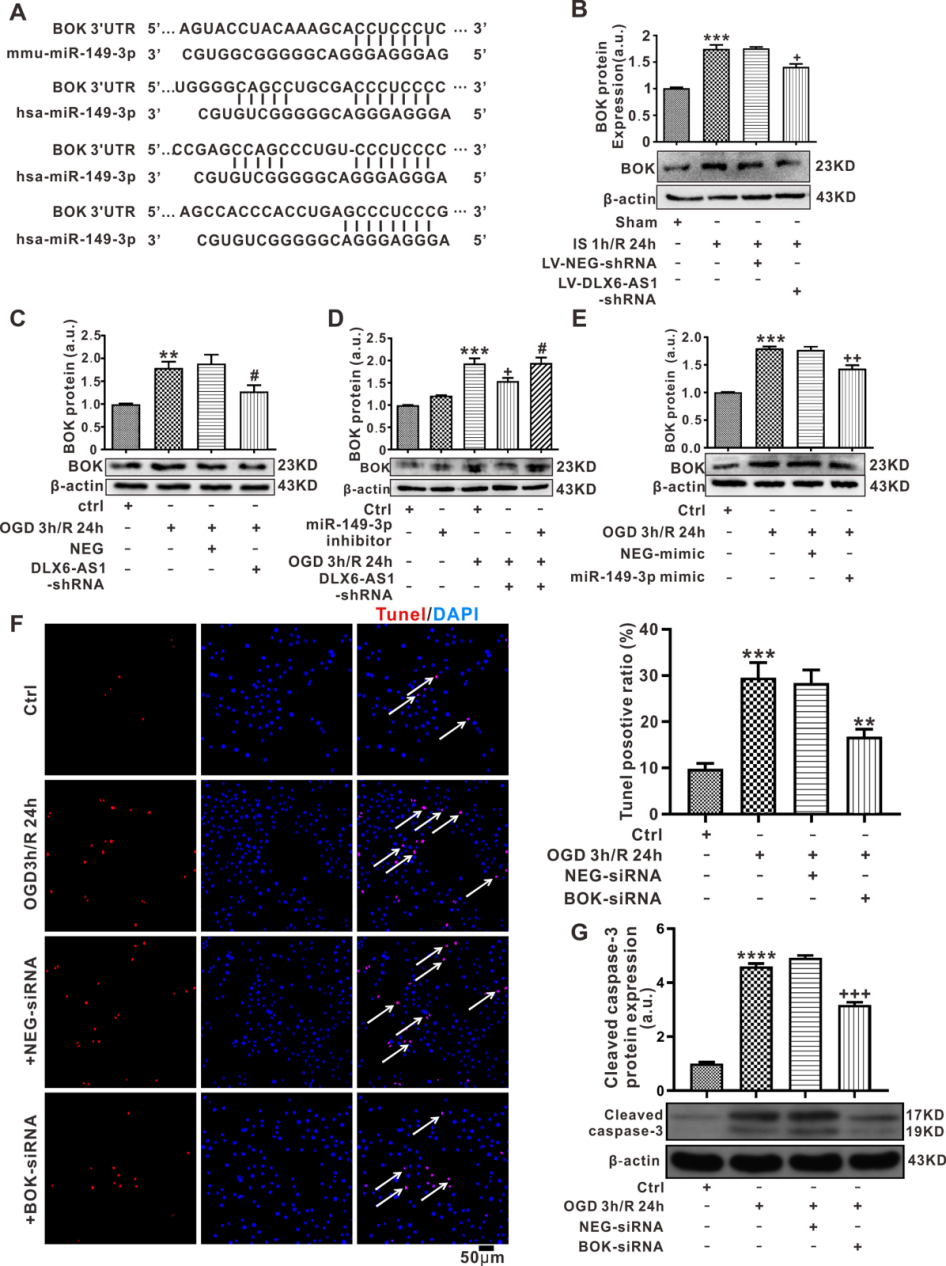
**BOK may be a target of miR-149-3p.** (**A**) The predicted binding sites of miR-149-3p and BOK by TargetScanVert. (**B**) BOK expression in the brain I/R model treated by LV-DLX6-AS1. (**C**–**E**) BOK protein expression following treatment with a DLX6-AS1 shRNA, miR-149-3p mimic or miR-149-3p inhibitor in N2a cells. (**F**) Representative images and statistics of TUNEL staining of N2a cells used to confirm apoptotic changes (100X). (**G**) Cleaved caspase-3 protein levels measured by western blotting. Values represent mean ± SEM (n = 3 in each group). **P < 0.01 vs Ctrl, ***P < 0.001 vs Ctrl or Sham or NEG; ^+^P < 0.05 vs IS 1 h/R 24 h + LV-NEG or OGD 3 h/R 24 h + NEG,^++^P < 0 .01 vs OGD 3 h/R 24 h + NEG-mimic or OGD 3 h/R 24 h + NEG-siRNA, ^+++^P < 0.001 vs OGD 3 h/R 24 h + NEG-siRNA; ^#^P < 0.05 vs OGD 3 h/R 24 h + NEG or OGD 3 h/R 24 h + DLX6-AS1-shRNA.

To explore the mechanism(s) of miR-149-3p actions in cerebral I/R, the target of miR-149-3p was predicted using TargetScanVert software. The results showed that a binding site for miR-149-3p was conserved in the 3’-UTR region of BOK in both Mus musculus and Homosapiens (Figure 6A). Alignment of the miR-149-3p sequence with BOK was performed to reveal that the BOK coding sequence may be a significant target for miR-149-3p. To verify BOK expression, we detected BOK protein expression both *in vivo* and *in vitro*. Interestingly, when compared to the sham or control groups, the expression of BOK protein was increased in the IS 1 h/R 24 h (1.82 ± 0.18, *P <* 0.001, Supplementary Figure 4A) and the OGD 3 h/R 24 h (1.63 ± 0.06, *P <* 0.01; Supplementary Figure 4B) groups.

For the purpose of verifying the regulatory mechanism of DLX6-AS1 with miR-149-3p on BOK, BOK protein expression was detected after silencing of DLX6-AS1 and before IS/R and OGD/R treatments. BOK protein was upregulated in cells with OGD/R or in brain tissue with cerebral I/R compared to the sham group. However, the reduction of DLX6-AS1 expression in LV-DLX6-AS1-shRNA+IS 1 h /R 24 h induced downregulation of BOK protein expression compared with that in the LV-NEG+IS 1 h /R 24 h group (Figure 6B). Similar results were found in the OGD/R model when transfected with DLX6-AS1-shRNA (Figure 6C). However, cells transfected with a miR-149-3p inhibitor exhibited upregulation of BOK protein expression compared with the DLX6-AS1-shRNA + OGD/R group (1.95 ± 0.12 *vs* 1.54 ± 0.07, *P <* 0.05, Figure 6D). Furthermore, overexpression of miR-149-3p following miR-149-3p-mimic transfection under the condition of OGD/R treatment obviously reduced BOK protein expression (1.43 ± 0.06, *P <* 0.01; Figure 6E) compared to that in NEG+OGD 3 h /R 24 h cells (1.77 ± 0.06). The results showed that DLX6-AS1 silencing inhibited BOK expression both *in vivo* and *in vitro*, which was reversed by a miR-149-3p inhibitor.

Next we determined the effect of BOK on neuronal apoptosis. We knocked down the expression of BOK in N2a cells via siRNA transfection and detected the effects of BOK on the apoptosis of N2a cells. BOK expression was successfully decreased by shRNA-1 (Supplementary Figure 4C). As expected, knockdown of BOK expression significantly inhibited OGD/R induced the ratio of TUNEL-positive cells (Figure 6F) and cleaved caspase-3 expression (Figure 6G) compared with that NEG treatment group. This finding was confirmed that BOK could promote OGD/R induced apoptosis. Taken together, these data suggest that DLX6-AS1 upregulates BOK expression by blocking miR-149-3p in OGD/R treatment N2a cells.

## DISCUSSION

With the advent of gene chips and gene sequencing, multiplexed research has found that lncRNAs fundamentally regulate the development of human diseases and may participate in stroke-induced apoptosis. DLX6-AS1 has mostly been recognized in terms of its regulation of cancer development by promoting cell proliferation and invasion [14]. Other research groups have suggested that DLX6-AS1 augments the carcinogenesis of glioma by interacting with endogenous sponging of miR-197-5p [6, 15]. Indeed, it is known that DLX6-AS1 is expressed in high levels in the brain but its potential role in stroke has remained unclear.

In the present study, we established a correlation between DLX6-AS1 expression and brain I/R impairment. Furthermore, silencing of LV-DLX6-AS1 inhibited I/R-induced apoptosis and ameliorated neurological dysfunction during the acute stages following I/R. Our Morris water maze and pole test results revealed that the decrease of DLX6-AS1 on the first day of training noticeably improved cognitive and motor functions after stroke. In previous research, it was reported that apoptosis of cerebral neurons mainly involved pathological changes in the peri-infarct region after the induction of global ischemia for a short period of time [16]. Our results have unequivocally demonstrated that DLX6-AS1 expression levels were upregulated and that the silencing of DLX6-AS1 reduced brain impairment induced by I/R in a mouse model. We also found that DLX6-AS1 could bind to miR-149-3p and inhibit the function of miR-149-3p on the target gene, BOK, resulting in neuronal apoptosis. Moreover, the silencing of DLX6-AS1 inhibited cerebral neuron apoptosis. These results indicated the protective effect of DLX6-AS1 silencing on neurons in the acute phase of I/R by inhibition neuronal apoptosis, but not through the promotion of the regeneration of neurons during rehabilitation.

As a primary mechanism of lncRNAs in disease development, lncRNAs are ceRNAs that act to compete with miRNAs for shared mRNAs binding [17]. DLX6-AS1 can bind miR-203a to promote the MMP-2 pathway in colorectal cancer [14]. In glioma carcinogenesis, DLX6-AS1 can endogenously sponge miR-197-5p to alleviate E2F1 and thus promote glioma development. Additionally, DLX6-AS1 may interact with miR-149-3p in both the mouse and human. Therefore, bioinformatics [14] was used to evaluate the ceRNA of DLX6-AS1 functions. Previous research has shown that when miR-149-3p is overexpressed it elicits apoptosis and promotes aggressiveness of cancer cells [18]. The potential actions of miR-149-3p stroke, however, remain to be elucidated. The results of our research have clearly demonstrated that the expression of miR-149-3p was lowered, and also in cells exposed to OGD/R. Moreover, an miR-149-3p mimic inhibited cellular apoptosis triggered by OGD/R. The data indicate that miR-149-3p actions are different and opposite on apoptosis and varies with different cell types. Transfection with an inhibitor of miR-149-3p reversed the function of DLX6-AS1 silencing of cells produced by OGD/R. These data strongly proof that miR-149-3p mediated the protective effect of DLX6-AS1 silencing in cerebral neurons by releasing miR-149-3p following ischemia.

BOK [19] can induce apoptosis of various types of cells through transcriptional and post-transcriptional activity [20] and can also regulate cell death by triggering the endoplasmic reticulum-related degradation pathway or by inducing apoptosis by the unfolded protein response when BAX/BAK is absent or under stress [21]. The data presented in the present study clearly showed that the levels of BOK were increased in the brain I/R model and OGD/R treated cells. The results of the research indicated that 3′-UTR of BOK is capable of binding to miR-149-3p. The expression of miR-149-3p was negatively correlated with impairments following ischemia. Additionally, DLX6-AS1 silencing inhibited BOK expression both *in vitro and in vivo*. A miR-149-3p inhibitor reversed the silencing effect of DLX6-AS1 on the expression of BOK. Transfection of N2a cells with BOK siRNA significantly inhibited OGD-R-induced apoptosis. These results suggested that BOK triggered DLX6-AS1 effects on apoptosis of cerebral neurons by sponging miR-149-3p. However, our investigation was limited, in that the promoter activation of BOK by the luciferase reporter assay was not detected.

In summary, it was clear that silencing of DLX6-AS1 inhibited apoptosis of ischemic cerebral neurons, an effect that may be mediated through the miR-149-3p/BOK signaling pathway. The research offers insights that might be helpful in developing effective therapies to promote the viability of cerebral neurons after brain I/R induced injury.

## MATERIALS AND METHODS

### Middle cerebral artery occlusion in mice

Adult male C57/BL6 mice (Certificate No: SCXK (Q) 2015-0018; weight range: 18–25 g; age: 2 months, n = 124) were purchased from the Hubei Provincial Center for Disease Control and Prevention. Female mice were not used to avoid any influences of sex steroids. C57/BL6 mice were housed in a 12-h light/ dark cycle in a climate-controlled room at the Experimental Animal Center of Wuhan University of Science and Technology. All mice were allowed free access to food and water before the procedure was performed under optimal conditions

(12/12 h light/dark cycle; humidity 60% ± 5%; temperature 22°C ± 3°C). Our institution’s local experimental ethics committee assessed and approved the proposed experiments and research protocols. Focal cerebral ischemia was induced by transient middle cerebral artery occlusion (tMCAO) for 60 min [22]. Briefly, mice were deeply anesthetized using a 1–2% isoflurane oxygen/nitrous oxide mixture in a ratio of 30% and 69% administered through a mask applied to the face; body temperature was maintained at 37 ± 0.3ºC with a small animal heating platform. The left common carotid as well as the external and internal carotid arteries were exposed and a silicone-coated 6-0 suture was routed from the stump of the external to the internal carotid artery until the lumen of the middle cerebral artery was reached. The distances from the bifurcation of the internal and external carotid artery to the middle cerebral artery was 10 ± 0.5 mm. Laser Doppler Flowmetry (Moor Instruments, UK) was used to establish that occlusion had been successfully achieved. The same procedure was used for sham-operated animals with the exception that the suture was routed along the internal carotid artery before being immediately withdrawn.

The sham group was comprised of 35 mice. In the LV-NEG–treated group, the mortality rate was 6.8% (3 of 44 mice) and the exclusion rate was 6.8% (3 of 44 mice). In the LV-DLX6-AS1–treated mice, the mortality rate was 6.8% (3 of 45 mice) and the exclusion rate was 4.4% (2 of 45 mice). Animals with hemorrhage of the middle cerebral artery or complications during surgery were excluded. Our institutional animal-care and use committee approved the research study.

### Assessments of mice behavior

The assessments were conduced 24 h after reperfusion. The Clark scoring method and the modified neurological severity score (mNSS) were used for behavioral analyses.

### Clark focal scales

A focal neurological scale (Clark focal test) was used for detailed evaluation of neurological defects, which displays a high correlation with the underlying infarct volume (Clark et al., 1997). The Clark focal test addresses body symmetry, gait, climbing, circling behavior, front limb symmetry, compulsory circling and whisker responses of mice. For each of the 7 categories assessed, the items were summed to provide a total focal score that ranged from 0 to 28.

### Modified neurological severity score (mNSS)

In the mNSS-points test, overall neurological function was evaluated following the guidelines of Chen et al [23]. The tests included the evaluation of motor responses (raising the mouse by the tail, placing the mouse on the floor), sensory (placing and proprioceptive tests), reflexive (reflex absence and abnormal movements) and balance (beam balance tests) deficits on a scale of 0 to 18 (0: normal score; 18: maximal deficit score).

### Morris water maze

The water maze task consisted of a circular water tank (120 cm in diameter and 60 cm in height) filled with opaque water (21–23°C), and a round platform (6 cm diameter), which was submerged 1 cm beneath the surface of the water at the center of the second quadrant. Before the start of hidden-platform training, mice were allowed to acclimate to the testing environment for 30 min. Hidden-platform training was carried out within 90 s over 5 consecutive days (6 sessions), with 4 trials in each session. If the mice failed to find the invisible platform within 60 s, they were guided to the platform and allowed to stand on it for 15 s. We then recorded escape latencies to find the hidden platform, swimming paths and swimming velocities. After 5 consecutive days of hidden-platform training, mice were allowed to rest for 1 day, and on day 6 a probe test was conducted. The platform was removed and the mice were allowed to search the pool for 60 s. The time spent in each quadrant was then analyzed. Data were traced through a TM-Vision video-tracking system (Chengdu Taimeng Software Co. Ltd, Chengdu, Sichuan Province, China).

### Pole test

As previously described [24], a pole test was conducted on the eighteenth experimental day and utilized to detect bradykinesia and/or the coordination of motor activity of the mice. Briefly, mice were positioned face upwards at the top of a wooden pole (1 cm diameter, 50 cm in length) and the time taken for a mouse to descend to the base recorded; the maximum permitted duration for this activity was 120 s. A total of 3 trials were conduced for each mouse and the median of the data across the trials calculated. Individual animals were ‘trained’ on 3 consecutive days before the experiment proper was conducted.

### Intracerebroventricular (icv) injection

C57/BL6 mice were anesthetized with 10% chloral hydrate and appropriately positioned on a stereotactic frame. LV-DLX6-AS1-down (LV-DLX6-AS1-shRNA1) and LV-negative-EGFP (LV-NEG) mice were given icv injections into the right lateral ventricle (10 μL syringe, rate 1 μL/min in a 4 μL total volume (Table 2), and were maintained in position for 5 min after the injection. The infusion coordinates were -1.0 mm lateral, -2.5 mm dorsal/ventral and -0.22 mm anterior/posterior to the bregma, respectively. After recovery from surgery mice were returned to their housing until the experimental endpoint was reached.

**Table 2:** Sequences.

**Name**	**Sequence**
Mouse DLX6-AS1 F	5’TGATCCTGGGGAGCTACGAA3’
Mouse DLX6-AS1 R	5’TTGAGCAACTTCCACGCTCA3’
Human DLX6-AS1 F	5’CCTTAGGGGTAGAAAGTAGGGC3’
Human DLX6-AS1R	5’CAAGCAGGAAGATCATGGGAG3’
Mouse BOK F	5’TGTCTTTGCAGCGGAGATCAT3’
Mouse BOK R	5’TCCCGGCCTAGTGCCTTAG3’
Mouse β-actin F	5’GGCTGTATTCCCCTCCATCG3’
Mouse β-actin R	5’CCAGTTGGTAACAATGCCATGT3’
Human β-actin F	5’GAGGGAAATCGTGCGTGAC3’
Human β-actin R	5’CTGGAAGGTGGACAGTGAG3’
Mouse DLX6-AS1 shRNA1	TTCCTAATGTAACAATGCGAA
Mouse DLX6-AS1 shRNA2	TAGAAGAGAACATTATGGAAT
Mouse DLX6-AS1 shRNA3	GGGGTCAGATCTATAGAAAGA
Mouse negative-shRNA	TTCTCCGAACGTGTCACGT
Mouse BOK-shRNA1	5’-GGCCACGCUCUGCAGCUUUTT-3’
	5’-AAAGCUGCAGAGCGUGGCCTT-3’
Mouse BOK-shRNA2	5’-GGCUCAGCCUGCCAUGGUUTT-3’
	5’-AAAGCUGCAGAGCGUGGCCTT-3’
Mouse BOK-shRNA3	5’-GGCCACGCUCUGCAGCUUUTT-3’
	5’-AAAGCUGCAGAGCGUGGCCTT-3’
Human DLX6-AS1 siRNA1	5’-GGAAAGAAGAGATTAGAAGAA-3’
Mouse miR-149-3p mimic	5’-GAGGGAGGGACGGGGGCGGUGC-3’
Mouse miR-149-3p inhibitor	5’-GCACCGCCCCCGUCCCUCCCUC-3’
	3’-AGUCACGUGAUGUCUUGAAAC-5’
Mouse NC mimic	5’-UUUGUACUACACAAAAGUACUG-3’
Mouse inhibitor mimic	5’-CAGUACUUUUGUGUAGUACAAA-3’

### 2, 3, 5-triphenyltetrazolium chloride (TTC) staining

Twenty-four hours after reperfusion each mouse was anesthetized, the brain quickly removed and sliced into 2 mm sections the coronal direction. Each section was incubated in 2% TTC for 30 min at 37°C (zero light) before being fixed in paraformaldehyde (4% solution). The ischemic region (pale) of each section was determined using Image J software (ver. 1.61) and the volume of the brain infarct evaluated thus: volume / total volume × 100% [10].

### Cresyl violet staining

Brain sections were post-fixed for 24 h at 4°C, and then immersed for 72 h in sucrose solution (30%, 4°C) to enable cryo-protection of the sections. To evaluate the degree of brain injury or atrophy, 20 μm specimen sections were stained with 0.1% cresyl violet. Next the ipsilateral and contralateral regional areas (hippocampus, striatum, corpus callosum, cortex, and midbrain) were examined (IMT i-Solution Inc, Canada).

### TUNEL labeling

Sections of brain and cultured cells were labeled with the TUNEL Bright Red Apoptosis Detection Kit System (Vazyme Biotech, US). Specimens were viewed on a CX31-32RFL confocal microscope. Cells undergoing apoptosis and the total number of cells were measured (Image J software). The apoptotic ratio was : apoptotic cells / total cells × 100%.

### Real-time RT-PCR

Total RNA was extracted with Trizol reagent (Invitrogen) according to the manufacturer’s instructions. The reverse transcription reaction was performed with 2 μg of total RNA using a First Strand cDNA Synthesis Kit (Thermo Fisher Scientific, K1622, MA, US). For PCR amplification, cDNA was amplified using SYBR Green Real-time PCR Master Mix (Takara, Shiga, Japan) and 0.4 μM of each primer pair. Amplification was carried out starting with an initial step of 5 min at 95°C, followed by 40 cycles of the amplification step (95°C for 10 s, 60°C for 30 s, and 70°C for 10 s) for mouse DLX6-AS1, human DLX6-AS1, mouse β-actin, human β-actin, miR-149-3p and U6 (the primers sequences are shown in Table 2). All amplification reactions for each specimen were carried out in triplicate and the means of the threshold cycles were used to interpolate curves using the Bio-Rad CFX manager (Bio-Rad, Richmond, CA, US). Results are expressed as the ratio of DLX6-AS1 to β-actin and miR-149-3p to U6 and the value of the control group was set to 1.

### Oxygen glucose deprivation/reperfusion model

SH-SY5Y and N2a cells were grown in DMEM high glucose solution with 1% penicillin-streptomycin and 10% fetal bovine serum in a humidified culture chamber (37°C, 5% CO_2_ atmosphere). Cells were seeded in 6-well plates (3 × 10^5^ cells per plate) and 24 h later the medium was replenished with serum-free glucose-free medium. Next, to induce oxygen glucose deprivation, plates were exposed to an atmosphere of 95% N_2_ and 5% CO_2_ at 37°C in an experimental chamber. Following 3 h oxygen glucose deprivation (OGD), normal medium was added to the plates. Cells were cultured under normoxic conditions for 0, 6, 12, 24 and 48 h at 37°C in a 5% CO_2_ atmosphere; control cells did not undergo OGD treatment.

### Cell transfection

For 24 h before OGD, cells were placed (3 × 10^5^ cells) in 6-well plates and transfected with DLX6-AS1-shRNA1, DLX6-AS1-shRNA2, DLX6-AS1-shRNA3 (see Table 2), an miR-149-3p inhibitor, an miR-149-3p mimic or a negative control by Lipofectamine 2000 (Invitrogen, US). After we detected the inhibitory effect of DLX6-AS1-shRNA, shRNA1 was chosen for the next experiments and construction of LV-DLX6-AS1 (*vide supra*). After 48 h, cells were harvested for RT-PCR and western blotting or were treated with OGD/R.

### Western blotting

The expression of caspase-3 and BOK proteins that had been cleaved were detected using western blotting. Brain tissues and cell specimens were extracted using a lysis buffer (RIPA, Applygen, Beijing) that had been supplemented with 0.1 mM phenylmethylsulfonyl fluoride to enable immunoblotting. The primary antibodies included rabbit anti-caspase-3 (1:1,000, 9662S, Cell Signaling Technology, MA, USA), rabbit anti-BOK (1:500) and mouse anti-β-actin (1:1,000, sc-47778, Santa Cruz Biotechnology, CA, USA). All antibodies were diluted with TBST buffer (0.1% Tween-20, 50 mM Tris-HCl, 150 mM NaCl, pH 7.4) and incubated at 4ºC overnight on PVDF membranes. Secondary antibodies conjugated with horseradish peroxidase (1:5,000, Abbkine Scientific Co. Ltd., US) were incubated for 90 min at ambient room temperature with the PVDF membranes. Signals were detected using an enhanced chemiluminescence reagent coupled to a Bio-Rad ChemiDoc MP instrument.

### RT-PCR

Expression levels of mRNA of mouse DLX6-AS1, human DLX6-AS1, mouse β-actin and human β-actin (primer sequences are shown in Table 2). miR-149-3p and U6 levels in brain sections and cells were analyzed. The methodology is documented in the supplementary materials section.

### Dual luciferase reporter gene assay

Assays were carried out as previously described [25]. Three binding sites on the DLX6-AS1 3′UTR were predicted to be regulated by miR-149a-3p (position1:98-106, position2:303-309, position3:490-498) and these sites were mutated via site-directed mutagenesis. The mutated (Mt) and wild-type (Wt) sequences of the 3'-UTR segments of DLX6-AS1 were inserted in to GV272 vectors (Genechem, Shanghai, China) to conduct luciferase reporter vectors. The sequence of miRNA-149-3p was inserted into the GV251 vectors (Genechem, Shanghai, China) to conduct miRNA-149-3p expression plasmid. For the reporter assays, the 293T cells were then placed in 24-well plates and 0.6 μg of GV251-mir-149-3p plasmid or the negative control was co-transfected with the 0.2 μg constructed DLX6-AS1 WT or MUT luciferase reporter vectors. The luciferase activity was measured using a luciferase assay kit (Promega, Madison, US) after transfection for 48 h according to the manufacturer's instructions. Experiments were repeated ≥ 3 times using different primary cells and plasmids.

### Statistical analysis

Data are reported as the mean ± SEM after ≥ 3 replicate measurements and analyzed using a *t*-test, one-way ANOVA and Tukey’s test. Statistical analyses were performed using Graph Pad Prism (ver. 5.0) and a *P*-value *<* 0.05 was considered to be a significant finding.

### Ethical approval

All applicable international, national, and/or institutional guidelines for the care and use of animals were followed. This article does not contain any studies with human participants performed by any of the authors.

## Supplementary Material

Supplementary Figures
