# Supplementary material for: Protective effect of DLX6-AS1 silencing against cerebral ischemia/reperfusion induced impairments
Source: Aging (Albany NY). 2020 Nov 18;12(22):23096–113. doi: 10.18632/aging.104070 (PMC7746362; doi:10.18632/aging.104070)
Supplement: Supplementary Figures [file aging-12-104070-s001..pdf]

SUPPLEMENTARY FIGURES

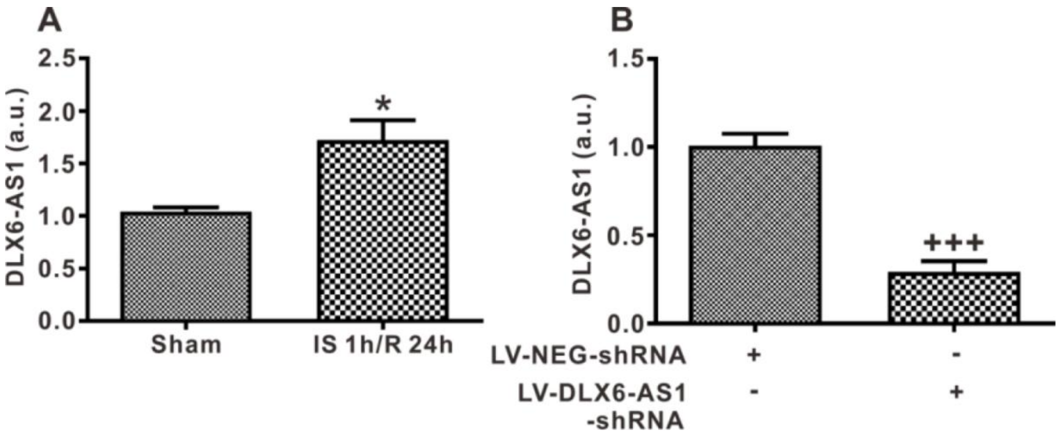

Supplementary Figure 1. DLX6-AS1 expression in stroke. (**A**) Normalized DLX6-AS1 *in vivo* expression levels upon I/R at reperfusion for 24 h. (**B**) DLX6-AS1 expression of mice icv injected with LV-DLX6-AS1-siRNA or LV-NEG seven days before MCAO. Values represent the mean ± SEM (n = 6 mice in each group). $^{*}P < 0.05$ *vs* sham, $^{+++}P < 0.001$ *vs* LV-NEG-shRNA.

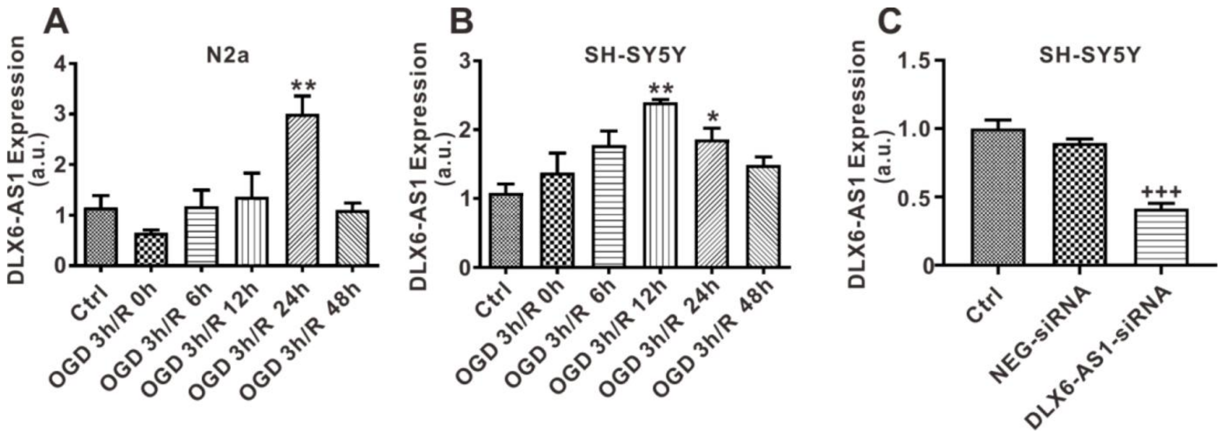

Supplementary Figure 2. DLX6-AS1 expression *in vivo* OGD/R. (**A**) Expression of DLX6-AS1 in N2a cells at different reperfusion times. (**B**) Expression of DLX6-AS1 in SH-SY5Y cells at different reperfusion times. (**C**) Expression of human DLX6-AS1 transfected by human DLX6-AS1-siRNA in SH-SY5Y. Values represent the mean ± SEM (n = 3 in each group). $^{*}P < 0.05$, $^{**}P < 0.01$ *vs* Ctrl; $^{+++}P < 0.001$ vs NEG-siRNA.

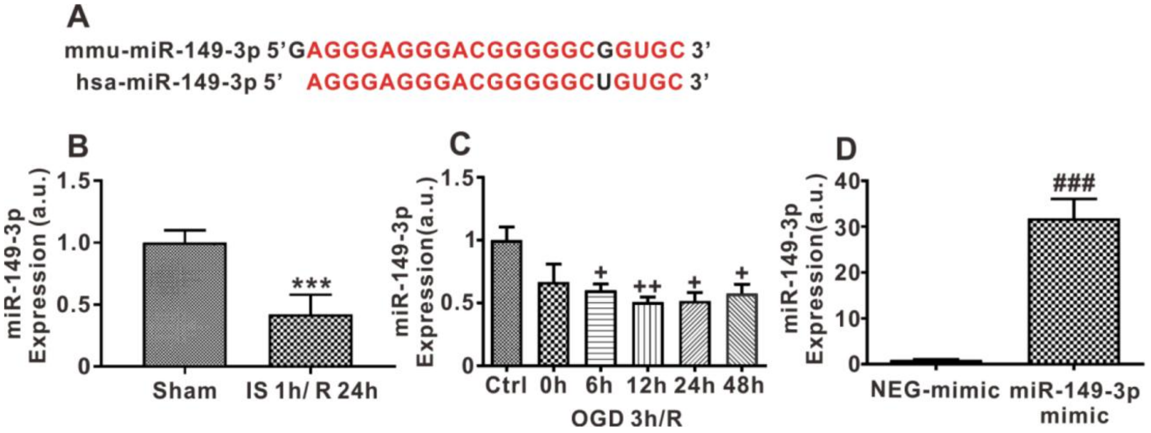

Supplementary Figure 3. miR-149-3p expression in stroke. (**A**) The sequences of mmu-miR-149-3p and hsa-miR-149-3p. (**B**) miR-149-3p expression of in the brains of mice treated by IS/R; (**C**) miR-149-3p expression in N2a cells treated by OGD/R; (**D**) A miR-149-3p mimic upregulates miR-149-3p expression. Values represent the mean ± SEM (n = 3–6 in each group). $^{***}P < 0.001$ *vs* Sham; $^{+}P < 0.05$, $^{++}P < 0.01$ *vs* Ctrl; $^{\#\#\#}P < 0.001$ *vs* NEG-mimic.

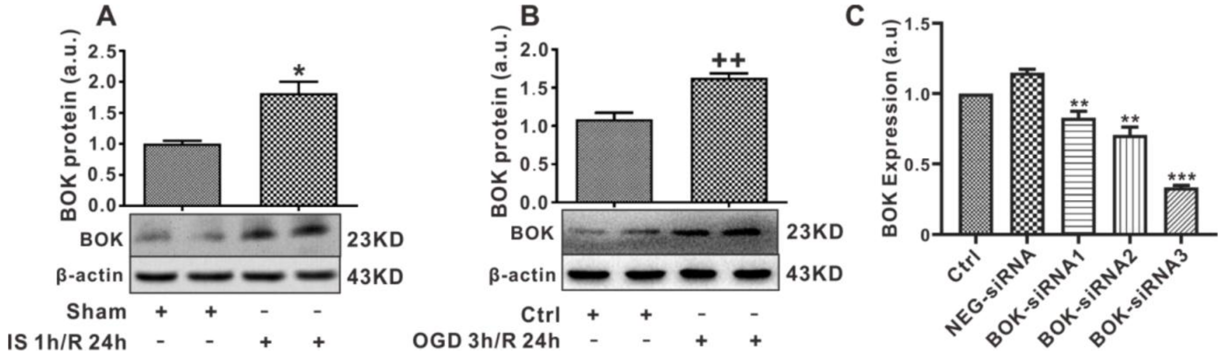

Supplementary Figure 4. BOK expression in stroke. (**A**) BOK expression in the brain I/R model. (**B**) BOK expression in N2a cells treated by OGD/R. (**C**) BOK mRNA expression of N2a cell treated by transfected with different BOK siRNA. Values represent the mean ± SEM (*n* = 3 in each group). $^{*}P < 0.05$ *vs* Sham; $^{++}P < 0.01$ *vs* Ctrl; $^{\#\#}P < 0.01$, $^{\#\#\#}P < 0.001$ *vs* NEG-siRNA.
